# The role of polyphenols in modern nutrition

**DOI:** 10.1111/nbu.12278

**Published:** 2017-08-15

**Authors:** G. Williamson

**Affiliations:** ^1^ University of Leeds Leeds UK

**Keywords:** cardiovascular disease, coffee, flavonoids, fruit, tea, type 2 diabetes

## Abstract

Polyphenols are found in plant‐based foods and beverages, notably apples, berries, citrus fruit, plums, broccoli, cocoa, tea and coffee and many others. There is substantial epidemiological evidence that a diet high in polyphenol‐rich fruit, vegetables, cocoa and beverages protects against developing cardiovascular disease and type 2 diabetes. The absorption and metabolism of these compounds have been well described and, for many, the gut microbiota play a critical role in absorption; taking into consideration the parent compound and the metabolites from colon bacteria catabolism, more than 80% of a dose can be absorbed and ultimately excreted in the urine. Common polyphenols in the diet are flavanols (cocoa, tea, apples, broad beans), flavanones (hesperidin in citrus fruit), hydroxycinnamates (coffee, many fruits), flavonols (quercetin in onions, apples and tea) and anthocyanins (berries). Many intervention studies, mechanistic *in vitro* data and epidemiological studies support a role for polyphenols against the development of chronic diseases. For example, flavanols decrease endothelial dysfunction, lower blood pressure and cholesterol, and modulate energy metabolism. Coffee and tea both reduce the risk of developing type 2 diabetes, through action of their constituent polyphenols. Despite extensive research, the exact mechanisms of action of polyphenols in the human body have not been decisively proven, but there is strong evidence that some targets such as nitric oxide metabolism, carbohydrate digestion and oxidative enzymes are important for health benefits. Consumption of polyphenols as healthy dietary components is consistent with the advice to eat five or more portions of fruit and vegetables per day, but it is currently difficult to recommend what ‘doses’ of specific polyphenols should be consumed to derive maximum benefit.

## Introduction

Modern nutrition is a multidisciplinary subject and draws from epidemiology, biochemistry, chemistry, behavioural science, biology, food science and medicine. It is necessary for human beings to consume food throughout their life, and so any biological effects will occur every day and have the potential to accumulate over a lifetime. Food choice, food availability, genetics, calories consumed and energy expenditure are additional factors which combine to make the subject of nutrition treacherous for the scientist hoping to develop hypotheses and prove theories. Biochemistry underlies all nutritional interactions, and understanding the pathways of metabolism, enzyme action and cellular regulation are essential for understanding how diet interacts with the body to affect health. Without this understanding, nutrition is a black box where scientific progress would be impossible. Nutrients comprise macronutrients (carbohydrate, fat and protein), which are digested and stored or used in the body, the micronutrients (vitamins and minerals), which are stored or temporarily retained in the body and are essential for facilitating basic biochemical processes, and the numerous other compounds that are not stored in the body and do not contribute directly to basic biochemical processes but which fine‐tune cells and protect against stress, helping to improve long‐term health in many different ways. Polyphenols belong to the latter and are a diverse group of molecules that are consumed in all diets (Scalbert & Williamson 2000). They originate only from plant‐based foods and have been termed non‐nutrients, plant secondary metabolites, phytonutrients, ‘antioxidants’, dietary bioactives and protective factors. Although there are a large number of chemical types, fortunately the number of polyphenols which are important in the diet is much smaller (Hertog *et al*. 1995).

## What are polyphenols? Nomenclature, classifications and occurrence in foods

‘Polyphenol’ is not a strict chemical term. Today, it is used to refer to flavonoids, tannins and phenolic acids and their various chemically modified or polymerised derivatives. The main classes of polyphenols in the UK diet are flavanols (including the catechins and tannins from tea), flavanones (mostly hesperidin from citrus fruit), flavonols (including quercetin from tea, apples and onions), hydroxycinnamic acids (phenolic acids, often called ‘chlorogenic acids’ and abundant in coffee and many fruit and vegetables) and anthocyanins (coloured polyphenols in fruit and vegetables). For more detailed information on the classes and distribution, the reader is referred to compositional databases such as Phenol‐Explorer (www.phenol-explorer.eu; Neveu *et al*. 2010) and to various reviews on intake amongst different populations (Perez‐Jimenez *et al*. 2011; Vogiatzoglou *et al*. 2014, 2015; Nishimuro *et al*. 2015; Pinto & Santos 2017). Most of the examples here will cover catechins and their oligomeric ‘tannins’, quercetin, hesperidin, anthocyanins and phenolic acids, which comprise the majority of the dietary polyphenols in Europe and the US (Table 1).

**Table 1:** Content of polyphenols in foods and beverages

Chemical class	Most common examples	Rich sources	Mean UK intake (mg/day)^a^	Comments on possible variations from the mean in individual diets
Flavanols	Catechins, gallocatechins (monomeric and oligomeric)	Tea (epicatechins, gallocatechins, theaflavins), cocoa (epicatechin, procyanidins), apples, broad beans (epicatechin)	590 (600)	Much higher in heavy tea drinkers
Flavanones	Hesperidin	Citrus fruit	25 (32)	Orange juice up to 500 mg/l
Flavonols	Quercetin, rutin	Tea, apples, onions	61 (40)	
Hydoxycinnamic acids	Chlorogenic acids (caffeoylquinic acids)	Coffee, chicory, artichoke, plum, pears	478 (517)	Up to 2000 mg/day in heavy coffee drinkers
Anthocyanins	Cyanidin	Berry fruits	20 (24)	A 100 g portion of blackberries contains ~170 mg anthocyanins

a Data from Yahya *et al*. (2016) (Leeds Wellbeing study) but intakes are dependent on individual diets and highly variable. Value in parentheses shows standard deviation.

## Absorption, metabolism and bioavailability

**Figure 1:**
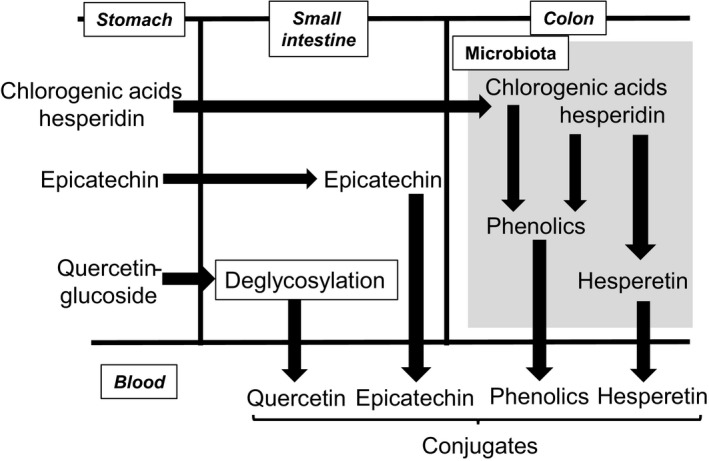
Highly simplified metabolic pathways involved in the absorption and metabolism of polyphenols. Phenolics = hydroxycinnamic acids. Chlorogenic acids and hesperidin are poorly absorbed intact, and so their attached organic acids and sugars, respectively, are efficiently removed by the gut microbiota. Deglycosylation is catalysed by brush border enzymes, especially lactase phlorizin hydrolase, and is most efficient when glucose is attached. Conjugates circulating post‐prandially in blood may be with methyl, glucuronic acid and/or sulphate groups.

Absorption and metabolism of polyphenols have been extensively studied and the biochemical pathways related to bioavailability are well understood for the most common classes (Fig. 1). The subject is, however, complicated by the extensive metabolism and the complex reactions catalysed by the gut microbiota in the colon. Classical bioavailability studies using radiolabelled compounds indicate that most tested polyphenols are well absorbed. For example, after consumption of either ^14^C‐labelled epicatechin by volunteers or ^14^C‐procyanidin B2 by rats, the proportion absorbed and subsequently appearing in the urine was over 80% (Stoupi *et al*. 2010; Ottaviani *et al*. 2016). This value includes absorption both of the parent molecule and of the lower molecular weight compounds produced by the gut microbiota. In addition, studies on ileostomist volunteers show that several polyphenols, such as quercetin, are well absorbed in the small intestine (Hollman *et al*. 1995) and studies using direct intestinal perfusion of volunteers show that enterocytes absorb and extensively metabolise several polyphenols (Petri *et al*. 2003; Actis‐Goretta *et al*. 2015). Nevertheless, the concentration reaching the blood is highly dependent on the parent polyphenol administered and it is likely that the concentrations of gut microbiota metabolites in the plasma generally exceed that of the parent compound (and its conjugates; Pimpao *et al*. 2015). The complex pathways of metabolism and conjugation have been well described in the literature, and most of the general principles are well understood (Del Rio *et al*. 2013; Fig. 1), but only limited information is available on the complex pathways of catabolism by gut microbiota (Woodward *et al*. 2011; Romo‐Vaquero *et al*. 2015; Williamson & Clifford 2017). In general, the peak concentrations of polyphenols in the blood post‐prandially are usually less than 1 μM, whereas, for gut catabolites, concentrations can well exceed this figure and are typically >10–100‐fold higher than the parent compound (Kay *et al*. 2009). Most of the circulating metabolites, both parent compound and gut microbiota metabolites, with some exceptions, are in the form of glucuronides and/or sulphates and may also be methylated (Fumeaux *et al*. 2010; Del Rio *et al*. 2013; Borges *et al*. 2016; Fig. 1). For example, several studies on bioavailability have clarified the pathways of absorption of epicatechin in detail. The predominant species in the plasma are 3′‐methyl‐epicatechin‐5‐*O*‐sulphate, epicatechin‐3′‐*O*‐sulphate and epicatechin‐3′‐*O*‐β‐D‐glucuronide, with epicatechin‐7‐*O*‐β‐D‐glucuronide additionally present in urine, and these have been quantified based on synthesised authentic standards (Actis‐Goretta *et al*. 2012; Ottaviani *et al*. 2012). Other detailed structures *in vivo* have also been reported for quercetin (Mullen *et al*. 2006), hesperidin (Leveques *et al*. 2012) and phenolic acids (Fumeaux *et al*. 2010). The extent of absorption is also influenced by numerous factors, such as chemical attachments on the polyphenol (Jaganath *et al*. 2006; Guo & Bruno 2015), solubility, processing (Neilson & Ferruzzi 2011; Cifuentes‐Gomez *et al*. 2015) and fat content (Cermak *et al*. 2003).

## Biological effects

Historically, polyphenols were mostly of interest to plant scientists as they play many roles in plants and form part of the class of secondary metabolites or phytochemicals. *In planta*, they protect against stresses such as UV light, deter attacks from pests and provide colour to attract certain insects. In the 1930s, one of the flavonoids, hesperidin, was proposed to be classified as a vitamin, vitamin P, and although this did not lead to an accepted classification, it was followed by numerous papers in the 1950s showing protective effects on the vascular system (*e.g*. Drezner *et al*. 1955). In the 1990s, polyphenols were classified as general antioxidants (Serafini *et al*. 1994) and this was originally thought to be a panacea to explain their global mechanism of action. However, the reality is much more complex and biological effects involve detailed biochemical interactions with pathways at the molecular level and, in this direction, much progress has been made in the last two decades. Although chemically polyphenols are antioxidants, this does not necessarily transfer to biological activity because any actions on the body depend both on bioavailability and cellular molecular targets. The overall effect on reducing the risk of disease is underpinned by epidemiology, where polyphenol‐rich foods and beverages are protective against development of some chronic diseases, especially type 2 diabetes and cardiovascular diseases (Yang *et al*. 2014a; Jumar & Schmieder 2016; Lee *et al*. 2016; Martin *et al*. 2016; Pang *et al*. 2016; Santos & Lima 2016). These studies are further supported by animal studies, *in vitro* cell mechanistic studies and a growing number of human intervention studies on both healthy and at risk volunteers, which are outlined below for selected classes of polyphenols.

## Human intervention studies on flavanols and flavanol‐rich foods such as cocoa

Many human intervention studies have been reported on cocoa, with a focus on its constituent flavanols ( –)‐epicatechin and its oligomers (procyanidins). In a review of 28 human intervention studies conducted between 2000 and 2007 on the effect of cocoa consumption (Cooper *et al*. 2008), the main outcomes were improved endothelial function, decreased susceptibility of low‐density lipoprotein (LDL) to oxidation, inhibition of platelet aggregation and activation, and decreased levels of F2‐isoprostanes. Further intervention studies have been reported and reviewed (Ding *et al*. 2006; Hooper *et al*. 2008; Buitrago‐Lopez *et al*. 2011; Jimenez *et al*. 2012; Ellam & Williamson 2013; Kerimi & Williamson 2015; Peluso *et al*. 2015; Martin *et al*. 2016; Vlachojannis *et al*. 2016). In many studies, regular consumption of cocoa, or a flavanol extract of cocoa, reduced blood pressure, blood cholesterol, F2‐isoprostanes and susceptibility of LDL to oxidation. These data indicate that the most significant changes remain as endothelial function, blood pressure and cholesterol levels, and, together with additional unpublished intervention studies, have enabled a claim on cocoa flavanols to be accepted by the European Food Safety Authority (EFSA NDA Panel 2014) on endothelium‐dependent vasodilation. Human intervention studies supporting the claim continue to be published (Heiss *et al*. 2015; Mastroiacovo *et al*. 2015). At the molecular level, many of the effects are mediated through interactions with nitric oxide metabolism in the blood vessel endothelium (Kerimi & Williamson 2015), improving endothelial dysfunction, increasing vasodilation and lowering blood pressure. All of these biomarkers indicate cardiovascular disease risk, hence providing evidence for the protective effect of flavanols against developing chronic cardiovascular conditions.

## Human intervention studies on flavanols and flavanol‐rich foods such as tea

**Figure 2:**
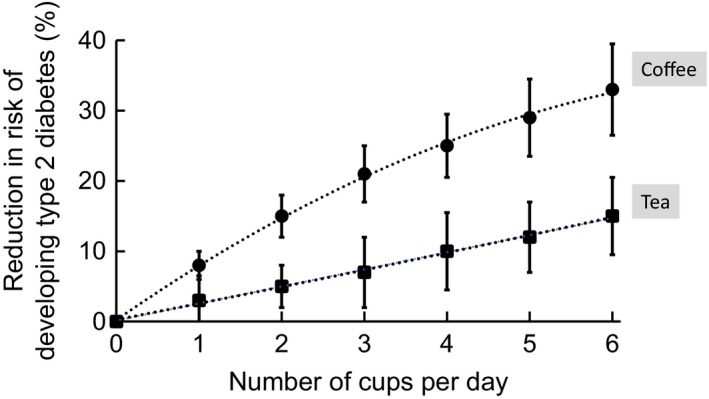
Relative risk reduction of developing type 2 diabetes with tea and coffee consumption. Plotted values for tea are from Yang *et al*. (2014b) and for coffee are from Ding *et al*. (2014). Reduction in risk calculated directly from [100‐relative risk (%)], and error bars indicate the upper and lower confidence intervals as reported in the cited papers. Data on coffee are consistent with studies by Bhupathiraju *et al*. (2013), who also showed that the reduction in risk was similar or even better (depending on cohort) for consumption of decaffeinated coffee compared to regular. Data on tea are supported by the meta‐analysis reported by Yang *et al*. (2014a). [Colour figure can be viewed at wileyonlinelibrary.com]

Tea is a rich source of catechins and gallocatechins. Green tea contains the ‘monomeric’ compounds as found in the plant but black tea contains mostly oxidised catechins, a chemically diverse group of polymerised molecules called theaflavins and thearubigins. Cocoa, green tea and black tea all contain various amounts of epicatechin, which is one of the most active polyphenols in these foods. It is not surprising, therefore, to find that some of the benefits associated with cocoa have also been found for tea, such as reduced risk of cardiovascular diseases (Pang *et al*. 2016). A Cochrane review summarises the effects of tea, after 3‐ to 6‐month intervention, as lowering blood pressure, lowering LDL‐cholesterol, not affecting high‐density lipoprotein (HDL)‐cholesterol and with no side effects, with a grading of the evidence as low/moderate quality (Santesso & Manheimer 2014). Regular consumption of tea is also associated with reduced risk of developing type 2 diabetes in meta‐analyses (Yang *et al*. 2014a, 2014b; Fig. 2). Despite numerous studies, the mechanisms of action in humans *in vivo* are still controversial and the associated anti‐diabetic activities have been ascribed to decreased rates of digestion and nutrient absorption (Hara & Honda 1990), such as attenuation of post‐prandial glucose spikes (Williamson 2013), activation of AMP‐activated protein kinase leading to changes in energy metabolism (Yang *et al*. 2016) and effects on gut microbiota (Yang *et al*. 2016).

## Human intervention studies on the flavonol, quercetin

Quercetin is mainly found in tea, apples and onions. All of these foods contain other biologically active components in addition to quercetin, and so some of the effects observed for tea described above could be partly ascribed to quercetin, and similarly for onions and apples. Regular consumption of the latter, for example, reduces the risk of type 2 diabetes according to a recent meta‐analysis (Guo *et al*. 2017). As quercetin is one of the most biologically active polyphenols *in vitro*, there have been several human intervention studies on the effects of pure quercetin. When given in the 4′‐*O*‐glucoside form, which is highly bioavailable, platelet aggregation and thrombus formation were reduced and coincided with the appearance of quercetin (metabolites) in plasma (Hubbard *et al*. 2004). When given chronically over 4 weeks, quercetin (as 3‐*O*‐glucoside, also bioavailable) improved endothelial function and reduced inflammation (Dower *et al*. 2015a), but did not affect flow‐mediated dilation or insulin resistance (Dower *et al*. 2015b). When given as aglycone (no sugars attached), which is much less bioavailable than the forms present in food (Shi & Williamson 2015), quercetin as a supplement decreased plasma uric acid in mildly hyperuricaemic males over a period of 4 weeks (Shi & Williamson 2016), but did not affect blood pressure in normal weight volunteers (Conquer *et al*. 1998; Egert *et al*. 2010b), platelet aggregation or serum cholesterol and triglyceride (Conquer *et al*. 1998), nor protect against oxidative stress after exercise (Nieman *et al*. 2007; McAnulty *et al*. 2008). When given acutely, quercetin reduced plasma endothelin‐1 (Loke *et al*. 2008), reduced blood pressure in overweight volunteers but only of a certain genotype (Egert *et al*. 2010a), but did not reduce urinary F2‐isoprostanes (Loke *et al*. 2008), nor blood pressure in normal weight volunteers (Egert *et al*. 2010b). When given in the form of onion skin extract, quercetin did not affect systemic inflammation in overweight women (Kim & Yim 2016), post‐prandial blood pressure, endothelial function, serum leptin nor adiponectin in overweight adults (Brull *et al*. 2016, 2017). These various studies suggest that quercetin exerts some effects in humans but the exact effect is highly dependent on the individual's metabolic and genetic status, and on the form given. As many of the studies gave relatively high doses, at this stage, more research is required to define the effect of quercetin from dietary sources.

## Human intervention studies on hydroxycinnamic acids and coffee

Many reviews and intervention studies have been reported on coffee and its constituent hydroxycinnamic acids (chlorogenic acids). The epidemiological evidence for a protective effect of coffee consumption against the risk of developing type 2 diabetes is very strong and shows a convincing dose‐dependent effect (Fig. 2). Data from systematic reviews and meta‐analyses show that the reduction in risk is ~8% for each cup of coffee consumed per day and that this is independent of whether the coffee is decaffeinated or not (Bhupathiraju *et al*. 2013; Ding *et al*. 2014). Some well‐publicised studies indicated that when coffee was boiled in the ‘Scandinavian’ way, there was an increase in plasma cholesterol (Zock *et al*. 1990), but these effects were due to cafestol and kahweol (Urgert *et al*. 1996). These components are not polyphenols and are present only in certain types of coffee (Urgert & Katan 1997). Intervention studies on coffee are more mixed and study designs are complicated by the fact that it is difficult to examine the effect of coffee consumption in regular coffee drinkers. The amount can either be increased to try to measure an additional effect of higher consumption, or coffee can be withdrawn for a period before reintroduction. In studies of the latter design, coffee over 4 weeks, after a 4‐week washout, attenuated DNA damage, reduced bodyweight and body fat (Bakuradze *et al*. 2011), and exerted beneficial effects on subclinical inflammation and HDL‐cholesterol (Kempf *et al*. 2010). Coffee consumption over 1 week has been reported to increase (Olthof *et al*. 2001) or not affect (Esposito *et al*. 2003) plasma homocysteine. Unfavourable effects of coffee on endothelial function (Buscemi *et al*. 2010) were in contrast to favourable effects of decaffeinated coffee (Buscemi *et al*. 2009). A more recent intervention where healthy volunteers received coffee for 2 months had no effect on markers of oxidation of DNA and lipids, plasma glucose and insulin, cholesterol, triglycerides, inflammatory markers, nor on blood pressure (Shaposhnikov *et al*. 2016). Nevertheless, a recent review on the effects of coffee from 26 intervention studies suggests that coffee consumption increases glutathione levels and protects against DNA damage, but with inconclusive effects on protein and lipid damage (Martini *et al*. 2016). Studies showing convincing mechanisms of action of coffee hydroxycinnamic acids are also lacking, but include effects on fat metabolism and the gut microbiota (Pan *et al*. 2016). The metabolism of chlorogenic acids from coffee is now well understood, involving critical action of gut microbiota, and knowledge on the plasma metabolites of chlorogenic acids should facilitate more mechanistic studies in the future. While the epidemiological data for coffee reducing the risk of type 2 diabetes are very convincing, the data are not yet backed up by well‐designed larger‐scale intervention studies and convincing mechanisms. Coffee consumption may also reduce the risk of colon cancer (Schmit *et al*. 2016), does not increase hypertension (Rhee *et al*. 2016) nor cancer risk (Arab 2010) and may be protective against cardiovascular disease (Crippa *et al*. 2014). Although many of the protective effects of coffee have been assigned to the constituent chlorogenic acids, there are very few intervention studies on pure chlorogenic acids in humans. A chlorogenic acid‐rich green coffee extract given for 12 weeks decreased both systolic and diastolic blood pressure, but did not affect body mass index nor pulse rate in patients with mild hypertension (Watanabe *et al*. 2006), and after consumption of an acute high dose of chlorogenic acid, there was no change in glucose tolerance via effects on incretin hormone secretion (Olthof *et al*. 2011). Clearly, one of the difficulties in making conclusions on the polyphenol component of coffee is separating out the effects of caffeine, which has substantial biological activity both positive and negative, and in addition, the presence of hydroxycinnamic acids may protect against some of the effects of caffeine.

## Supplements or not?

Supplements have the advantage of delivering a suitably active dose but also have several drawbacks. Often polyphenols act with other nutrients and a key example is where polyphenols already present in food slow down the rate of carbohydrate digestion to blunt post‐prandial glucose spikes (Williamson 2013). If taken without food, a supplement would be unable to have any effect on this parameter and so the ideal situation would be to consume the polyphenol with food (*i.e*. in its natural state). In addition, supplements may have modified bioavailability and can also discourage the consumption of a ‘healthy’ diet in favour of supplementing a poor diet. The use of plant food supplements (botanical supplements) in Europe has been reviewed (Vargas‐Murga *et al*. 2011), and in a survey of people from six countries (Finland, Germany, Italy, Romania, Spain and the UK), almost 20% of those asked consumed plant food supplements, containing a total of more than 490 different ingredients, with the most common ones being *Ginkgo biloba*,* Oenothera biennis* (Evening primrose) and *Cynara scolymus* (Artichoke) (Garcia‐Alvarez *et al*. 2014).

## Future perspectives

Most micronutrients such as vitamins and minerals have an officially approved daily intake recommended value, with these varying between countries and regions. Although initially proposed for polyphenols (Williamson & Holst 2008), differences between polyphenols and vitamins suggest that a somewhat different approach is needed. This is based on the assumption that a sufficient dose for an effect is needed every time of consumption and that, unlike minerals and vitamins, the active component is not stored or temporarily retained in the body. For example, a food containing 10 mg of a certain mineral will contribute 10% to a daily intake recommended value of 100 mg, and 10 doses will therefore constitute sufficient daily intake. On the other hand, for components with a proposed beneficial effect, such as cocoa flavanols, but not stored in the body, the magnitude of the effect is dependent on dose. The critical question is how much is needed for the smallest biologically significant effect, which would then be effective and observable over a suitable period of time. This is a truly important question in this area if we consider a situation where the daily effect of an acute dose is not measurable by current biomarker technology, but the effect (not the actual compound) builds up and becomes apparent over a period of weeks, months or even years. Let us consider that the amount necessary for a given effect by a hypothetical flavonoid is 10 mg per day and that the effect is apparent after 2 months. If we give a food containing a ‘dose’ of 5 mg, this may not elicit this threshold effect and so the dose could be considered as ‘wasted’, at least in terms of affecting the desired biomarker. Therefore, one proposition is that the recommended daily intake for these compounds cannot be achieved by administering 2 × 5 mg doses separately, only by a single 10 mg bolus. This needs to be considered and scientifically evaluated before any compound‐specific recommendations on intake can be made.

## Conflict of interest

GW has recently, or currently, received research funding from Nestle and Florida Department of Citrus, conducted consultancy for Nutrilite, US, and Suntory, UK, and was a partner in the EU framework 7 project BACCHUS with involvement of several companies including small‐to‐medium enterprises (SMEs).
